# The antinociceptive effect of 4-substituted derivatives of 5-(4-chlorophenyl)-2-(morpholin-4-ylmethyl)-2,4-dihydro-3*H*-1,2,4-triazole-3-thione in mice

**DOI:** 10.1007/s00210-013-0938-0

**Published:** 2013-12-11

**Authors:** Listos Joanna, Sylwia Talarek, Jolanta Orzelska, Sylwia Fidecka, Monika Wujec, Tomasz Plech

**Affiliations:** Medical University, Lublin, Poland

**Keywords:** Antinociceptive activity, The hot plate test, The writhing test, Behavioral tests

## Abstract

The aim of the present experiments was to examine the antinociceptive activity of 4-substituted derivatives of 5-(4-chlorophenyl)-2-(morpholin-4-ylmethyl)-2,4-dihydro-3*H*-1,2,4-triazole-3-thione in mice. The compounds were synthesized using the so-called Mannich reaction and their structures were confirmed using IR and 1H-NMR spectra. The antinociceptive activity was investigated in two behavioral tests: the hot plate test and the writhing test. For preliminary estimation of other behavioral effects, the locomotor activity of mice, the motor coordination in the rota-rod test, and the myorelaxation in the chimney test were also studied. The changes in body temperature of animals were also recorded. We demonstrated that all examined compounds produced antinociceptive effect, both in the hot plate test and in the writhing test, without impact on the motor coordination and myorelaxation of animals. The pharmacological effect of all drugs has been developed within 60 min after administration of drugs; and in two cases (T-103 and T-104), it has been a short-lasting effect (up to 90 min). Two compounds (T-100 and T-102) also inhibited the locomotor activity of animals. T-104 induced the changes in body temperature of mice. Generally, we demonstrated that combination of two different heterocyclic systems (morpholine and 1,2,4-triazole) might be beneficial for reduction of nociception.

## Introduction

Pain is an unpleasant sensory and emotional experience that serves to alert an individual to actual or potential tissue damage. This damage can be caused by exposure to noxious chemical, mechanical, or thermal stimuli or by the presence of a pathologic process (tumor, muscle spasm, inflammation, nerve damage etc.; Mao 2009; Rabow and Pantilat 2014). In order to select and apply appropriate therapy, pain is distinguished by different types, such as somatic, visceral, neuropathic pain, etc. Each kind of pain is associated with different mechanisms, like inflammation, noxious stimulus, or nerve damage; thus, each situation requires different treatment schemes (Mao 2009; Rabow and Pantilat 2014).

Pain, perceived as suffering syndrome, should be alleviated as uncontrolled pain may induce further pathological changes, including shock. Generally, there are two groups of analgesics that are commonly used in the therapy of somatic and visceral pain (Turk et al. 2011). Non-steroidal anti-inflammatory drugs and paracetamol are recommended for the therapy of mild to moderate pains, while opioids, the oldest analgesics, are mainly used to alleviate intense acute and severe chronic pains, often post-operative and cancer-related. Neuropathic pain is controlled by other drugs, such as tricyclic antidepressants or anti-seizure drugs (Dworkin et al. 2010). Unfortunately, typical analgesic drugs produce adverse effects. For example, non-steroidal anti-inflammatory drugs (NSAIDs) may induce peptic ulcers, heart failure and hypertension, renal dysfunctions, and allergy, while opioids may produce the state of tolerance and dependence or, at higher doses, respiratory depression (Grosser et al. 2011; Yaksh and Wallace 2011).

Thus, in the twenty-first century, pain therapy leaves much to be desired and is rather far from ideal. Therefore, searching for new drugs which could improve the quality of pain therapy seems to be most purposeful and firmly justified. Several reports have demonstrated that the search for new analgesics has already been undertaken and the antinociceptive activity of different derivatives of 1,2,4-triazole has repeatedly been demonstrated (Gowda et al. 2011; Hussein et al. 2011; Uzgören-Baran et al. 2012; Vijesh et al. 2013). A very similar effect has also been induced by pyrazoline (Joshi et al. 2010) and morpholine derivatives (Ahmadi et al. 2011; Xu et al. 2008).

Therefore, the aim of the present experiments was to examine new compounds from the point of view of their antinociceptive activity. All the studied compounds were 4-substituted derivatives of 5-(4-chlorophenyl)-2-(morpholin-4-ylmethyl)-2,4-dihydro-3*H*-1,2,4-triazole-3-thione. Thus, all drugs are the combination of 1,2,4-triazole and morpholine groups which support their significance in pain control. The antinociceptive activity of the examined drugs was investigated in mice in the hot plate test and the writhing test, i.e., two generally accepted behavioral tests that are widely used in experimental neuropsychopharmacology to evaluate the antinociceptive effects of drugs. The hot-plate test used for evaluation of the centrally acting analgesics (Bastia et al. 2002; Grecksch et al. 2006; Liu et al. 2003, Thomsen et al. 2007) is a neurogenic-modulated model that produces, at constant temperature, two types of behavioral response (paw licking and jumping), both of which are considered to be supraspinally integrated. In turn, the writhing test is a chemical method used to induce pain of the peripheral origin by injection of irritant agents, such as acetic acid, in mice (Bastia et al. 2002; Liu et al. 2003; Reichert et al. 2001). Therefore, both tests were applied in the described experiments to extend knowledge on antinociceptive effects of the examined drugs and to recognize their influence on the centrally and peripherally acting stimuli. Also, the locomotor activity of mice, the motor coordination in rota-rod test, the miorelaxant effect in chimney test, and the changes in body temperature were studied in mice for preliminary estimation of other behavioral effects of new drugs.

## Materials and methods

### Chemistry

All the used reagents and solvents were purchased from Alfa Aesar (Ward Hill, USA) and Merck Co. (Darmstadt, Germany). Melting points were determined by a Fisher-Johns apparatus (Fisher Scientific, Schwerte, Germany) and were uncorrected. The ^1^H NMR spectra were recorded on a 250-MHz Bruker Avance spectrometer (Bruker BioSpin GmbH, Germany), using TMS as an internal standard and DMSO-d_6_ or CDCl_3_ as solvent. IR spectra were obtained on a Perkin-Elmer 1725X FTIR spectrophotometer. Elemental analyses were performed on an AMZ 851 CHX analyser (PG, Gdansk, Poland) and the obtained results were within ±0.4 % of their theoretical value.

### General procedures for the synthesis of educts

Respective thiosemicarbazide and 1,2,4-triazole-3-thione derivatives were obtained according to the method described earlier (Plech et al. 2011). A solution of 10 mmol (1.70 g) of 4-chlorobenzoic acid hydrazide and equimolar amount of appropriate aryl isothiocyanate in 25 ml of anhydrous EtOH was heated under reflux for 15 min. Next, the ethanolic solution was cooled and the solid formed was filtered off, washed with diethyl ether, dried, and recrystallized from EtOH.

*1*-(*4*-*Chlorobenzoyl*)-*4*-(*2*,*4*-*dichlorophenyl*)*thiosemicarbazide*

Yield: 90 % (3.36 g). Elemental analysis data for C_14_H_10_Cl_3_N_3_OS (374.67); calculated/found (%): C 44.88/44.70, H 2.69/2.85, N 11.22/11.08. All information on the compound may be retrieved in the CAS database (CAS Registry Number: 891643-33-7).

*4*-(*4*-*Bromophenyl*)-*1*-(*4*-*chlorobenzoyl*)*thiosemicarbazide*

Yield: 92 % (3.54 g). Elemental analysis data for C_14_H_11_BrClN_3_OS (384.68); calculated/found (%): C 43.71/43.79, H 2.88/2.67, N 10.92/10.86. All information on the compound may be retrieved in the CAS database (CAS Registry Number: 356576-31-3).

*1*-(*4*-*Chlorobenzoyl*)-*4*-[*4*-*chloro*-*3*-(*trifluoromethyl*)*phenyl*]*thiosemicarbazide*

Yield: 79 % (3.22 g). ^1^H NMR (DMSO-*d*_6_): 7.03–7.74 (m, 7H, Ar-H), 10.04, 10.57, 11.56 (3 s, 3*H*, 3NH). Elemental analysis data for C_15_H_10_Cl_2_F_3_N_3_OS (408.22); calculated/found (%): C 44.13/44.02, H 2.47/2.67, N 10.29/10.18.

*1*-(*4*-*Chlorobenzoyl*)-*4*-(*3*,*4*-*dichlorophenyl*)*thiosemicarbazide*

Yield: 82 % (3.07 g). Elemental analysis data for C_14_H_10_Cl_3_N_3_OS (374.67); calculated/found (%): C 44.88/44.75, H 2.69/2.78, N 11.22/11.34. All information on the compound may be retrieved in the CAS database (CAS Registry Number: 891643-96-2).

*1*-(*4*-*Chlorobenzoyl*)-*4*-(*3*-*chlorophenyl*)*thiosemicarbazide*

Yield: 85 % (2.89 g). Elemental analysis data for C_14_H_11_Cl_2_N_3_OS (340.23); calculated/found (%): C 49.42/49.58, H 3.26/3.45, N 12.35/12.19. All information on the compound may be retrieved in the CAS database (CAS Registry Number: 443298-33-7).

In order to obtain 1,2,4-triazole-3-thiones, a solution of respective thiosemicarbazide derivative (10 mmol) in 2 % NaOH was heated under reflux for 2 h. After cooling, the mixture was neutralized with 3 M HCl. The precipitate formed was filtered off and washed with distilled water. The compounds were recrystallized from EtOH.

*4*-(*2*,*4*-*Dichlorophenyl*)-*5*-(*4*-*chlorophenyl*)-*2*,*4*-*dihydro*-*3H*-*1*,*2*,*4*-*triazole*-*3*-*thione*

Yield: 83 % (2.96 g), m.p. 238-240 °C, ^1^H NMR (DMSO-*d*_6_): 7.39 (d, 2H, Ar-H, *J* = 8.6 Hz), 7.55 (d, 2H, Ar-H, *J* = 8.6 Hz), 7.70–7.95 (m, 3*H*, Ar-H), 14.39 (s, 1H, NH). Elemental analysis data for C_14_H_8_Cl_3_N_3_S (356.66); calculated/found (%): C 47.15/47.13, H 2.26/2.38, N 11.78/11.60.

*4*-(*4*-*Bromophenyl*)-*5*-(*4*-*chlorophenyl*)-*2*,*4*-*dihydro*-*3H*-*1*,*2*,*4*-*triazole*-*3*-*thione*

Yield: 81 % (2.97 g). Elemental analysis data for C_14_H_9_BrClN_3_S (366.66); calculated/found (%): C 45.86/45.70, H 2.47/2.60, N 11.46/11.37. All information on the compound may be retrieved in the CAS database (CAS Registry Number: 537017-82-6).

*4*-[*4*-*Chloro*-*3*-(*trifluoromethyl*)*phenyl*]-*5*-(*4*-*chlorophenyl*)-*2*,*4*-*dihydro*-*3H*-*1*,*2*,*4*-*triazole*-*3*-*thione*

Yield: 76 % (2.96 g), m.p. 242-245 °C, ^1^H NMR (DMSO-*d*_6_): 6.93–7.56 (m, 7H, Ar-H), 14.11 (s, 1H, NH). Elemental analysis data for C_15_H_8_Cl_2_F_3_N_3_S (390.21); calculated/found (%): C 46.17/46.28, H 2.07/2.00, N 10.77/10.85.

*4*-(*3*,*4*-*Dichlorophenyl*)-*5*-(*4*-*chlorophenyl*)-*2*,*4*-*dihydro*-*3H*-*1*,*2*,*4*-*triazole*-*3*-*thione*

Yield: 86 % (3.07 g), m.p. 280–282 °C, ^1^H NMR (DMSO-*d*_6_): 7.06–7.68 (m, 7H, Ar-H), 14.08 (s, 1H, NH). Elemental analysis data for C_14_H_8_Cl_3_N_3_S (356.66); calculated/found (%): C 47.15/47.26, H 2.26/2.09, N 11.78/11.64.

*4*-(*3*-*Chlorophenyl*)-*5*-(*4*-*chlorophenyl*)-*2*,*4*-*dihydro*-*3H*-*1*,*2*,*4*-*triazole*-*3*-*thione*

Yield: 80 % (2.58 g), m.p. 272–273 °C, ^1^H NMR (DMSO-*d*_6_): 7.10–7.61 (m, 8H, Ar-H), 14.13 (s, 1H, NH). Elemental analysis data for C_14_H_9_Cl_2_N_3_S (322.21); calculated/found (%): C 52.19/52.30, H 2.82/2.76, N 13.04/12.90.

### General procedure for the synthesis of compounds named as T-100–T-104

To a solution of corresponding 5-(4-chlorophenyl)-4-substituted-2,4-dihydro-3*H*-1,2,4-triazole-3-thione (10 mmol) in anhydrous ethanol (35 ml) equimolar amounts of morpholine and formaldehyde (37 %) were added. The obtained mixture was stirred at room temperature for 2 h. Next, distilled water (5 ml) was added and the precipitate formed was filtered off and recrystallized from ethanol. Spectral and physicochemical data of compounds T-100–T-104 are listed in Table 1.

**Table 1:** Yields, melting points, and spectral data for compounds T-100–T-104

	Yield (%)	m.p. (°C)	^1^H-NMR	IR	Molecular formula/weight	Elemental analysis (calculated/found)
T-100	78	180–182	2.95 (t, 4H, 2 × CH_2_, *J* = 4.6 Hz), 3.64 (t, 4H, 2 × CH_2_, *J* = 4.6 Hz), 5.25 (s, 2H, CH_2_), 7.10–7.87 (m, 7H, Ar-H)	3056, 2901, 2840, 1597, 1320	C_19_H_17_Cl_3_N_4_OS 455.78	C 50.07/50.23 H 3.76/3.86 N 12.29/12.10
T-101	77	Data presented in Plech et al. (2012)				
T-102	70	221–223	2.94 (t, 4H, 2 × CH_2_, *J* = 4.8 Hz), 3.76 (t, 4H, 2 × CH_2_, *J* = 4.8 Hz), 5.26 (s, 2H, CH_2_), 7.22–7.73 (m, 7H, Ar-H)	3104, 3029, 2875, 1607, 1323	C_20_H_17_Cl_2_F_3_N_4_OS 489.34	C 49.09/48.93 H 3.50/3.64 N 11.45/11.49
T-103	82	201–202	2.93 (t, 4H, 2 × CH_2_, *J* = 4.6 Hz), 3.75 (t, 4H, 2 × CH_2_, *J* = 4.7 Hz), 5.25 (s, 2H, CH_2_), 7.12–7.64	3029, 2923, 1613, 1319	C_19_H_17_Cl_3_N_4_OS 455.78	C 50.07/50.11 (m, 7H, Ar-H) H 3.76/3.60 N 12.29/12.41
T-104	75	189–192	2.92 (t, 4H, 2 × CH_2_, *J* = 4.7 Hz), 3.76 (t, 4H, 2 × CH_2_, *J* = 4.8 Hz), 5.29 (s, 2H, CH_2_), 7.08–7.71 (m, 8H, Ar-H)	3087,3023, 2945, 1611, 1312	C_19_H_18_Cl_2_N_4_OS 421.34	C 54.16/54.28 H 4.31/4.11 N 13.30/13.45

### Pharmacology

#### Animals

The experiments were carried out on male albino Swiss mice (20–30 g). The animals were kept 8–10 per cage at room temperature of 22 ± 1 °C, on natural day–night cycle (spring). Standard food (Murigran pellets, Bacutil, Motycz) and tap water were freely available. All the experiments were made between 9 a.m. and 2 p.m. After 1 week of adaptation and handling, the animals were divided into groups (10–14 animals/group) and prepared for the tests.

The study was performed according to the National Institute of Health Guidelines for the Care and Use of Laboratory Animals and the European Community Council Directive for Care and Use of Laboratory Animals and was approved by local ethics committee (The Medical University of Lublin Committee on the Use and Care of Animals).

#### Drugs

Before behavioral experiments, all the examined compounds were suspended in a little amount (3–4 drops) of Tween 80 and then diluted in saline. All the drugs were administered intraperitoneally (ip), except the new compounds in the writhing test that were subcutaneously (sc) injected. All the drugs were applied in volume of 10.0 ml/kg.

The control animals received same volumes of saline at the respective time before the test.

The acute toxicity of the studied compounds was assessed in mice, according to the Litchfield and Wilcoxon method (1949), as ED_50_ calculated for mortality within 48 h. In the pharmacological experiments, each compound was injected in doses equivalent to 0.1 and 0.05 ED_50_ (Table 2).

**Table 2:** ED50 values and doses of the drugs used in behavioral experiments

Drug	ED_50_ value (mg/kg)	The doses of drug used in behavioral experiments (mg/kg)
T-100	100	5 and 10
T-101	250	12.5 and 25
T-102	2,000	100 and 200
T-103	2,000	100 and 200
T-104	2,000	100 and 200

The lack of the significant changes in time spending on the rotating rod and in the chimney confirms that all of the studied compounds did not influence on the motor coordination and myorelaxation of mice

#### Behavioral experiments

The antinociceptive activity of the examined compounds was studied in two tests:

The hot plate test was performed according to the method described by Woolfe and MacDonald, (1944). In that test, the animals were placed on hot (55 °C), metal plate. The time to the first reaction (licking or jumping) of mice to a nociceptive stimulus was measured. The test was performed 30, 60 and 90 min after injection of a studied compound. The cut-off time was set to 60 s to avoid severe pain or tissue damage.

The writhing test (Siegmund et al. 1957) is a chemical method, generally used to study peripheral pain, induced by ip injection of irritant acetic acid (0.6 %), as a nociceptive stimulus, inducing characteristic writhing episodes. In that test, all the examined compounds were administered (sc) 50 min before injection of acetic acid (ip), and 5 min after acetic acid administration, the number of writhing episodes was recorded for a period of 10 min.

Other parameters were also assessed in behavioral tests, in order to extend the knowledge about new compounds, including the locomotor activity, the motor coordination, the myorelaxation and the changes in body temperature of mice. The locomotor activity in mice was measured in round actometer cages (32 cm in diameter, Multiserv, Lublin, Poland), which were kept in a sound-attenuated experimental room. The cages were equipped with one row of infrared light-sensitive photocells located 1 cm above the floor. The locomotor activity was measured 30 min after compound injection for a total period of 30 min (Rump and Kleinrok 1982). The motor coordination of mice was assessed in the rota-rod test (Ataner Zakład Uslugowy Elektroniki Inż K. Fic, Lublin, Poland) and the miorelaxant effects were measured in the chimney test. The rota-rod test (Dunham and Miya 1957) assesses the ability of animals to keep balance on a rotating rod (20 mm diameter) during 60 s. The rod revolves with constant speed of 18 rpm. The time period that the mice were spent on the rotating rod, was measured and recorded. The chimney test (Listos et al. 2010) evaluates the ability of mice to go backwards, vertically from the tube. Accordingly, the time period that the mice were spent in a tube (maximum 60 s) was measured and recorded. The tube was made of Plexiglas with rough surface (30 mm diameter and 25 cm length). Both tests were performed 60 min after injection (ip) of the examined drugs.

Before the rota-rod test and the chimney test, the animals had been trained: each mouse was placed on the rod and in the tube for 3 min. The number of trials for each mouse was unlimited. Then, consequently, only those animals were approved that were able to stay on the rotating rod for 60 s or were able to leave the chimney without much problem (up to 15 s).

The body temperature of animals was monitored using an electronic thermometer with a rectal probe during the total period 240 min (60 min before and 180 min after drug administration). The average of the first two measurements (60 and 30 min before drug injection) was determined as an initial temperature (Δi). After drug administration the final temperature (Δf) was measured six times every 30 min (up to 180 min) in each mice. The changes in body temperature (Δ) were calculated according to the formula: Δ = Δf − Δi. The changes in body temperature were studied after administration of high doses of all the studied compounds.

### Statistical analysis

All behavioral results, presented in the figures and tables as mean ± SEM, were statistically calculated using the one-way analysis of variance (ANOVA). *Post hoc* comparisons were carried out by means of the Tukey test. The probability (*p*) value of 0.05 or less was considered as statistically significant. Each group of animals consisted of 8–10 mice.

## Results

**Schemes 1:**
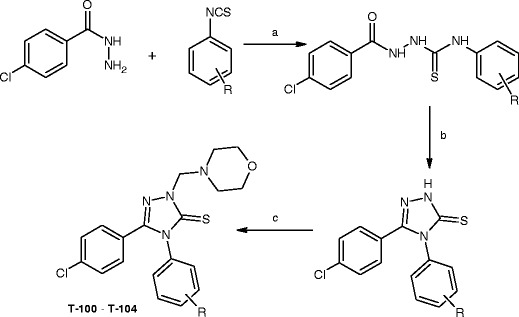
Synthetic route to target compounds T-100–T-104. Reagents and conditions: **a** EtOH, reflux, 15 min; **b** 2 % NaOH, reflux, 2 h; and **c** HCHO, morpholine, EtOH, 2 h, rt. List of substituents: *R* = 2,4-diCl (T-100), 4-Br (T-101), 3-CF_3_-4-Cl (T-102), 3,4-diCl (T-103), 3-Cl (T-104)

The synthesis pathway, leading to compounds T-100–T-104 was depicted in Scheme 1. The structure and purity of the compounds were characterized, using IR and ^1^H-NMR spectra, combined with an elemental analysis. In ^1^H-NMR spectra, a sharp singlet, integrated for two protons of the methylene group (linking the morpholine and the 1,2,4-triazole rings) was visible in the range of 5.25–5.29 ppm. Chemical shifts of the morpholine hydrogens occurred between 2.92 and 3.76 ppm, while the aromatic protons resonated as multiplets at 7.08–7.87 ppm. In IR spectra, absorption bands, characteristic for C=S, C=N, –CH_2_– groups, were visible in ranges of 1,312–1,323, 1,597–1,613, and 2,840–2,945 cm^−1^, respectively.

### Effects of the new derivatives in the hot plate test

**Fig. 1:**
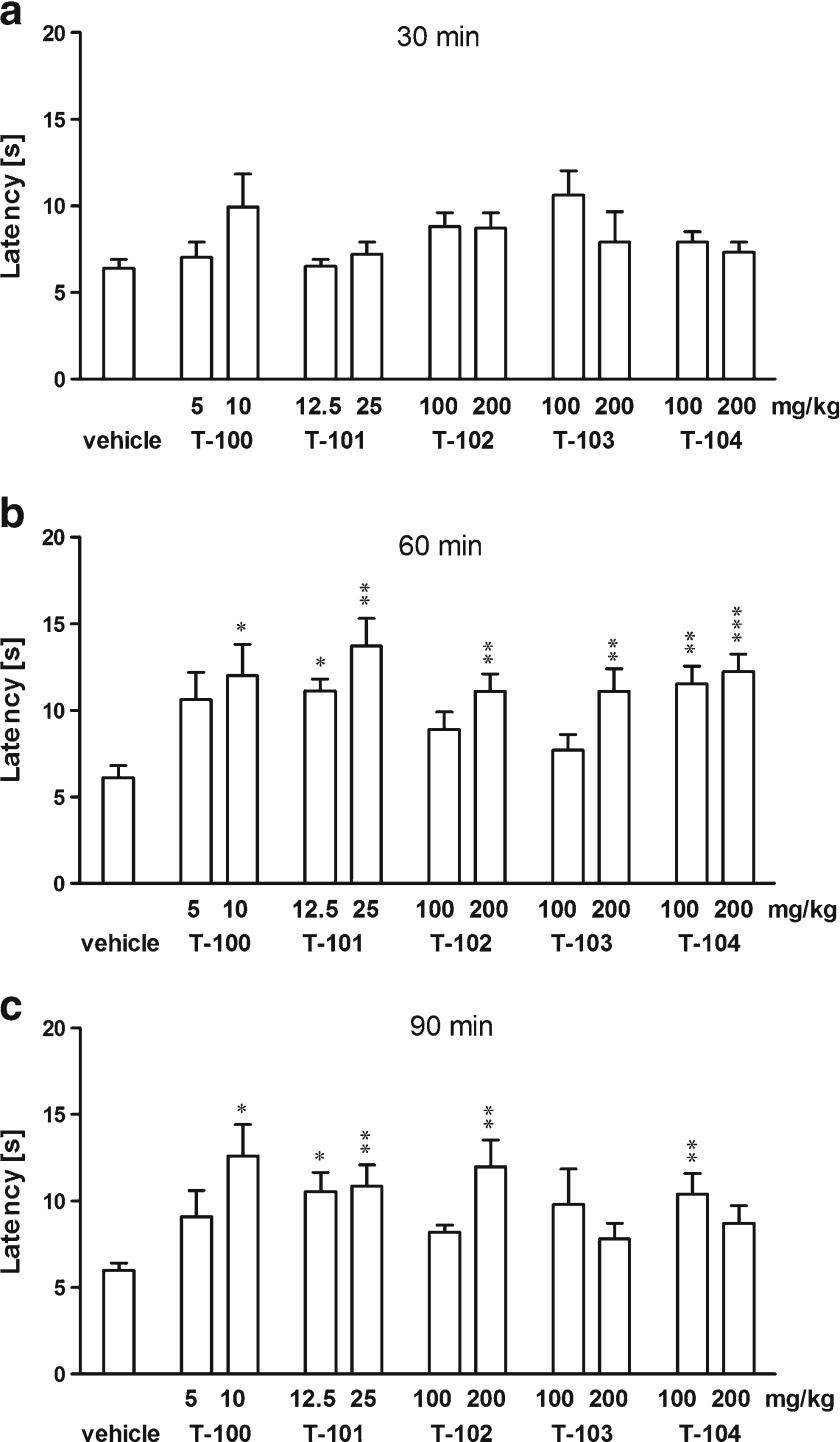
Effects of T-100 (5, 10 mg/kg), T-101 (12.5, 25 mg/kg), T-102 (100, 200 mg/kg), T-103 (100, 200 mg/kg), and T-104 (100, 200 mg/kg) on response latency in the hot- plate test in mice. Reaction latency values were recorded at 30 (**a**), 60 (**b**), and 90 min (**c**) following the administration of drugs. Data are presented as mean ± SEM. ***p < 0.001, **p < 0.01, *p < 0.05 vs vehicle-treated group (Tukey’s test)

Thirty minutes after the injection of examined drugs, the one-way ANOVA did not show any significant effect, while after 60 min significant changes were demonstrated: T-100: *F*_2,25_ = 4.17, *p* = 0.0285; T-101: *F*_2,24_ = 7.401, *p* = 0.0003; T-102: *F*_2,24_ = 8.006, *p* = 0.0024; T-103: *F*_2,23_ = 6.921, *p* = 0.0049; T-104: *F*_2,23_ = 13.64, *p* = 0.0002. In turn, after 90 min from the injections the one-way analysis demonstrated significant changes in three cases only: T-100: *F*_2,25_ = 5.157, *p* = 0.0141; T-101: *F*_2,24_ = 6.992, *p* = 0.0045; T-102: *F*_2,24_ = 7.552, *p* = 0.0032. A *post hoc* analysis showed that 60 min after the administration of examined drugs, significant and dose-dependent changes were induced by either dose of T-101 (12.5 and 25 mg/kg—*p* < 0.05, *p* < 0.01, respectively) and T-104 (100 and 200 mg/kg—*p* < 0.01 and *p* < 0.001, respectively). In the case of the other drugs, significant effects were demonstrated only after high doses: T-100 (10 mg/kg—*p* < 0.05), T-102 (200 mg/kg—*p* < 0.01), T-103 (200 mg/kg—*p* < 0.05). In 90 min after the administration of T-100, T-101, and T-102, the antinociceptive effects were similar to the previous effect; however, the effects of T-103 and T-104 were completely diminished (Fig. 1).

### Effects of the new derivatives in the writhing test

**Fig. 2:**
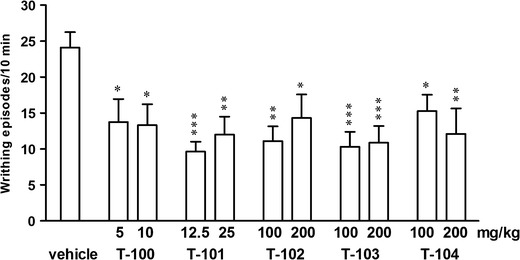
Effects of T-100 (5, 10 mg/kg), T-101 (12.5, 25 mg/kg), T-102 (100, 200 mg/kg), T-103 (100, 200 mg/kg), and T-104 (100, 200 mg/kg) on acetic acid-induced abdominal writhing in mice. Data are presented as mean ± SEM of writhing episodes. ***p < 0.001, **p < 0.01, *p < 0.05 vs. the vehicle-treated group (Tukey’s test)

The one-way analysis revealed significant changes after the administration of all the examined compounds: T-100: *F*_2,24_ = 5.568, *p* = 0.011; T-101: *F*_2,27_ = 12.29, *p* = 0.000; T-102: *F*_2,29_ = 6.614, *p* = 0.0046; T-103: *F*_2,25_ = 13.34, *p* = 0.0001; T-104: *F*_2,27_ = 5.719, *p* = 0.009. A *post hoc* analysis demonstrated significant antinociceptive effect after each dose of the administered: T-100 (5 and 10 mg/kg—*p* < 0.05); T-101 (12.5 and 25 mg/kg—*p* < 0.001 and *p* < 0.01, respectively); T-102 (100 and 200 mg/kg—*p* < 0.01 and *p* < 0.05, respectively); T-103 (100 and 200 mg/kg—*p* < 0.001); T-104 (100 and 200 mg/kg—*p* < 0.05 and *p* < 0.01, respectively; Fig. 2).

### Effects of the new derivatives in other tests (locomotor activity, rota-rod test, chimney test, the changes in body temperature)

**Fig. 3:**
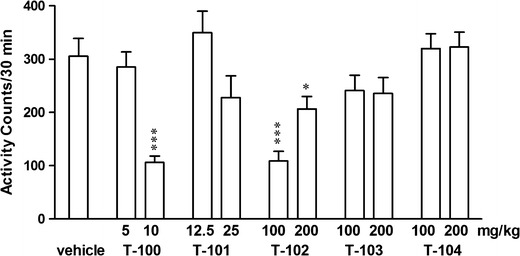
Effects of T-100 (5, 10 mg/kg), T-101 (12.5, 25 mg/kg), T-102 (100, 200 mg/kg), T-103 (100, 200 mg/kg), and T-104 (100, 200 mg/kg) on locomotor activity in mice. Data are presented as mean ± SEM of beam breaks recorded for 30 min. ***p < 0.001, *p < 0.05 vs. the vehicle-treated group (Tukey’s test)

The one-way ANOVA showed significant changes in the locomotor activity of studied mice after two drugs: T-100 and T-102: *F*_2,27_ = 19.12, *p* < 0.001 and *F*_2,24_ = 12.6, *p* < 0.0002, respectively. A *post hoc* analysis demonstrated that high dose of T-100 (*p* < 0.001) and both doses of T-102 (*p* < 0.001 and *p* < 0.05, respectively) significantly inhibited the locomotor activity in studied animals, while the other three drugs (T-101, T-103, and T-104) did not produce any significant effects on the locomotor activity of the mice (Fig. 3). No significant changes in motor coordination of mice in the rota-rod test and in myorelaxation in the chimney test (Table 3).

**Table 3:** The results obtained in the rota-rod test and in the chimney test

Drug	Dose of drug (mg/kg)	The time on the rotating rod (in seconds)	The time in the chimney (in seconds)
Vehicle	–	47.000 ± 4.575	9.733 ± 0.887
T-100	5	58.010 ± 1.990	9.178 ± 0.906
	10	38.140 ± 5.595	12.690 ± 1.954
T-101	12.5	51.820 ± 5.658	9.731 ± 0.857
	25	52.700 ± 4.686	12.17 ± 2.199
T-102	100	46.140 ± 5.179	10.030 ± 0.969
	200	38.570 ± 6.311	9.613 ± 1.048
T-103	100	31.660 ± 7.488	6.640 ± 0.885
	200	29.180 ± 7.710	7.880 ± 0.930
T-104	100	35.250 ± 7.063	7.960 ± 0.934
	200	50.200 ± 5.430	7.676 ± 1.063

The significant changes in body temperature in mice were observed only after administration of T-104: *F*_11,90_ = 12, *p* < 0.0001. *Post hoc* analysis confirmed that T-104 induced the significant reduction of body temperature in comparison with control animals. The changes were observed 30 min (*p* < 0.05), 60 and 90 min (*p* < 0.001), 120 min (*p* < 0.05), and 150 and 180 min (*p* < 0.01) after drug administration. One-way ANOVA did not show the significant changes in body temperature of animals after administration of: T-100, T-101, T-102, and T-103 (Table 4).

**Table 4:** The changes in body temperature (°C) after administration of high dose of the studied compounds

Drug	Changes in body temperature [Δ°C]					
	30’	60’	90’	120’	150’	180’
Vehicle	−0.5 ± 0.187	−0.112 ± 0.279	0.537 ± 0.316	0.157 ± 0.193	0.200 ± 0.197	0.287 ± 0.222
T-100 (10 mg/kg)	−0.087 ± 0.386	−0.550 ± 0.931	−0.237 ± 0.525	−0.537 ± 0.2605	0.125 ± 0.295	0.100 ± 0.271
T-101 (25 mg/kg)	−0.987 ± 0.405	−0.225 ± 0.392	0.014 ± 0.392	0.742 ± 0.249	0.312 ± 0.430	0.225 ± 0.452
T-102 (200 mg/kg)	−1.000 ± 0.126	−0.757 ± 0.212	−0.050 ± 0.056	−0.400 ± 0.151	−0.500 ± 0.264	−0.350 ± 0.396
T-103 (200 mg/kg)	−1.325 ± 0.464	−0.8375 ± 0.368	−0.317 ± 0.290	0.214 ± 0.251	−0.050 ± 0.172	−0.150 ± 0.189
T-104 (200 mg/kg)	−1.829 ± 0.458*	−1.586 ± 0.199***	−1.371 ± 0.300***	−1.214 ± 0.218*	−1.225 ± 0.076**	−1.257 ± 0.097**

***p < 0.001, **p < 0.01, and *p < 0.05 vs. the vehicle-treated group (Tukey’s test)

## Discussion

In the described experiment, the antinociceptive activity of five 4-substituted derivatives of 5-(4-chlorophenyl)-2-(morpholin-4-ylmethyl)-2,4-dihydro-3*H*-1,2,4-triazole-3-thione was studied. We demonstrated antinociceptive effects in all the examined compounds, both in the hot plate test and the writhing test, without impact on the motor coordination (in rota-rod test) or myorelaxation (in the chimney test) of animals. Pharmacological effects of all the applied developed within 60 min after their administration and, in two cases (T-103 and T-104), their short-lasting effects (up to 90 min) were observed. The two compounds (T-100 and T-102) suppressed the locomotor activity of the studied animals and the other—T-104—induced the significant reduction of body temperature in mice.

The obtained results clearly demonstrate that both T-100, containing 2,4-dichlorophenyl as substituent, and T-102, containing 4-chloro-(3-trifluoromethyl)phenyl, require further studies to define their significance with regards to the central nervous system functions. Both drugs produced significant effects in the anti-nociceptive tests. The reduction in the locomotor activity of the studied mice, could, however, been attributed to drug-induced sedation or other toxic effects, which may preclude the use of these compounds as potential drugs. On the other hand, it is possible that dose reduction of the drugs may also diminish their toxicity. Taking into account the clear, antinociceptive effects in both tests, and the significant reduction in the locomotor activity, it can be supposed that both compounds produce their pharmacological effect *via* some receptors in the central nervous system, e.g., the opioid receptors. All such speculations, however, need further studies to unveil their mechanisms.

T-103, which contains 3,4-dichlorophenyl as substituent, produced stronger peripheral antinociceptive effect in the writhing test vs. the central effects in the hot plate test. It may then be assumed that the drug produced mainly peripheral antinociceptive effects, although poor penetration of the compound across the blood-brain barrier cannot be excluded, either. Similar properties were observed in the case of T-104 that contains 3-chlorophenyl as substituent. Both drugs (T-103 and T-104) produced short-lasting effects, as after 90 min from their injections and no effect was observed in the hot plate test. T-104 significantly reduced the body temperature. The lack of changes in the locomotor activity, the motor coordination, and myorelaxation of the studied mice suggests that especially T-103 may be non-toxic compound, thus prompting further studies to confirm its safety and efficacy.

The most promising effect was observed in the case of T-101 that contains 4-bromophenyl as substituent. Strong, dose-dependent and long-lasting antinociceptive effects were observed in the hot plate test and clearly antinociceptive effects were also demonstrated in the writhing test. Moreover, the compound did not induce any changes in the locomotor activity, the motor coordination or myorelaxation of the studied mice. Thus, T-101 seems to be a relatively safe agent, although it requires further studies to confirm its safety and efficacy.

The search for new analgesic drugs is well grounded. Admittedly, two groups of analgesics are used in clinical practice: weaker (NSIADs) and stronger analgesics (opioids); however, all of them produce some adverse effects that limit their free usage. Opioids also induce the state of tolerance and the state of dependence (Grosser et al., 2011, Yaksh and Wallace, 2011). Therefore, any experiments, which may lead to formulate new analgesic drugs, give chance to obtain a new medicinal agent or a group of medicinal agents with different functionalities and improved pharmacological properties, substantially helping to extend the range of pain therapies, their effectiveness, and safety. The presented results show only a fragment of investigations that would be necessary to identify and understand the complete pharmacological profile of the examined compounds. Accordingly, a series of biochemical and pharmacokinetic experiments are planned to extend the range of our knowledge on the new medicinal agents. Although further studies are necessary to identify all the properties of derivatives that contain the combination of morpholine and 1,2,4-triazole groups, T-101, as long-lasting drug, and T-103 and T-104, as short-lasting compounds, may be considered as the most promising for further experiments.

## Conclusions

In summary, a synthesis and an identification of five, 4-substituted derivatives of 5-(4-chlorophenyl)-2-(morpholin-4-ylmethyl)-2,4-dihydro-3*H*-1,2,4-triazole-3-thione have been presented and discussed. Potential antinociceptive activities of all the studied drugs were demonstrated in two behavioral tests (the hot-plate test and the writhing test) and their effect on locomotor activity, motor coordination, myorelaxation and changes in body temperature in mice was shown. All the examined compounds showed some biological activity that confirms the beneficial effect of combining two different heterocyclic systems (morpholine and 1,2,4-triazole) in nociception. Further experiments are necessary to specify other properties of the present compounds.
